# cGMP: a unique 2nd messenger molecule – recent developments in cGMP research and development

**DOI:** 10.1007/s00210-019-01779-z

**Published:** 2019-12-18

**Authors:** Andreas Friebe, Peter Sandner, Achim Schmidtko

**Affiliations:** 1 Institute of Physiology, University of Würzburg, Röntgenring 9, D-97070 Würzburg, Germany; 2 Drug Discovery, Bayer AG, Aprather Weg 18a, D-42096 Wuppertal, Germany and Institute of Pharmacology, Hannover Medical School, Carl-Neuberg-Str. 1, D-30625 Hannover, Germany; 3 Institute of Pharmacology and Clinical Pharmacy, Goethe University, Max-von-Laue-Str. 9, D-60438 Frankfurt am Main, Germany

**Keywords:** cGMP, Guanylyl cyclases, Phosphodiesterases, Nitric oxide, Natriuretic peptides, sGC stimulators, Riociguat, Praliciguat, Vericiguat, sGC activators

## Abstract

Cyclic guanosine monophosphate (cGMP) is a unique second messenger molecule formed in different cell types and tissues. cGMP targets a variety of downstream effector molecules and, thus, elicits a very broad variety of cellular effects. Its production is triggered by stimulation of either soluble guanylyl cyclase (sGC) or particulate guanylyl cyclase (pGC); both enzymes exist in different isoforms. cGMP-induced effects are regulated by endogenous receptor ligands such as nitric oxide (NO) and natriuretic peptides (NPs). Depending on the distribution of sGC and pGC and the formation of ligands, this pathway regulates not only the cardiovascular system but also the kidney, lung, liver, and brain function; in addition, the cGMP pathway is involved in the pathogenesis of fibrosis, inflammation, or neurodegeneration and may also play a role in infectious diseases such as malaria. Moreover, new pharmacological approaches are being developed which target sGC- and pGC-dependent pathways for the treatment of various diseases. Therefore, it is of key interest to understand this pathway from scratch, beginning with the molecular basis of cGMP generation, the structure and function of both guanylyl cyclases and cGMP downstream targets; research efforts also focus on the subsequent signaling cascades, their potential crosstalk, and also the translational and, ultimately, the clinical implications of cGMP modulation. This review tries to summarize the contributions to the “9th International cGMP Conference on cGMP Generators, Effectors and Therapeutic Implications” held in Mainz in 2019. Presented data will be discussed and extended also in light of recent landmark findings and ongoing activities in the field of preclinical and clinical cGMP research.

## Introduction

Nitric oxide (NO) and natriuretic peptides (NPs) are important messenger molecules. Their binding to soluble guanylyl cyclase (sGC) and particulate guanylyl cyclase (pGC), respectively, increases the formation of cGMP which is a unique second messenger. cGMP-driven activation of protein kinases, ion channels, or phosphodiesterases (PDEs) causes a broad variety of physiological responses whereas dysregulation can result in severe pathologies. Thus, understanding the mechanisms of cGMP generation and downstream signaling cascades is fundamental for the comprehension of molecular physiology and pathophysiology as well as for the development of drugs within these pathways that, as demonstrated in recent years, have already substantial therapeutic implications. An overview of sGC and pGC signaling, the physiology, pathophysiology as well as basic and clinical pharmacology of cGMP has been presented in recent monographies and reviews (Schmidt et al. (eds.) 2009; Krieg and Lukowsky 2013; Buglioni and Burnett Jr. 2016; Schlossmann (ed.), Schlossmann 2018; Kuhn 2016; Sandner et al. 2019).

In brief, the production of cGMP is increased either after stimulation of sGC by nitric oxide (NO) formed from L-arginine by NO synthases (NOS) or after activation of pGCs by specific peptides. Several effectors of cGMP have been identified, most importantly cGMP-regulated protein kinases (abbreviated as cGKs or PKGs). These kinases phosphorylate downstream target proteins which mediate cGMP effects, e.g., by decreasing the intracellular calcium concentration. cGMP signaling is abrogated by cGMP hydrolysis via PDEs and cGMP export via multidrug resistance proteins (also referred to as ABC transporters). However, these signaling cascades seem to be highly compartmentalized in individual cell types; in addition, the expression of the members of these pathways can vary between tissues and in healthy or disease states which is currently being investigated with various sensors and was also discussed in this meeting. Based on the differential expression of sGC, pGC, or regulatory PDEs, their subcellular localization and the potential crosstalk between this and other pathways, the net effects of pharmacological intervention into the cGMP cascade are difficult to predict.

**Fig. 1:**
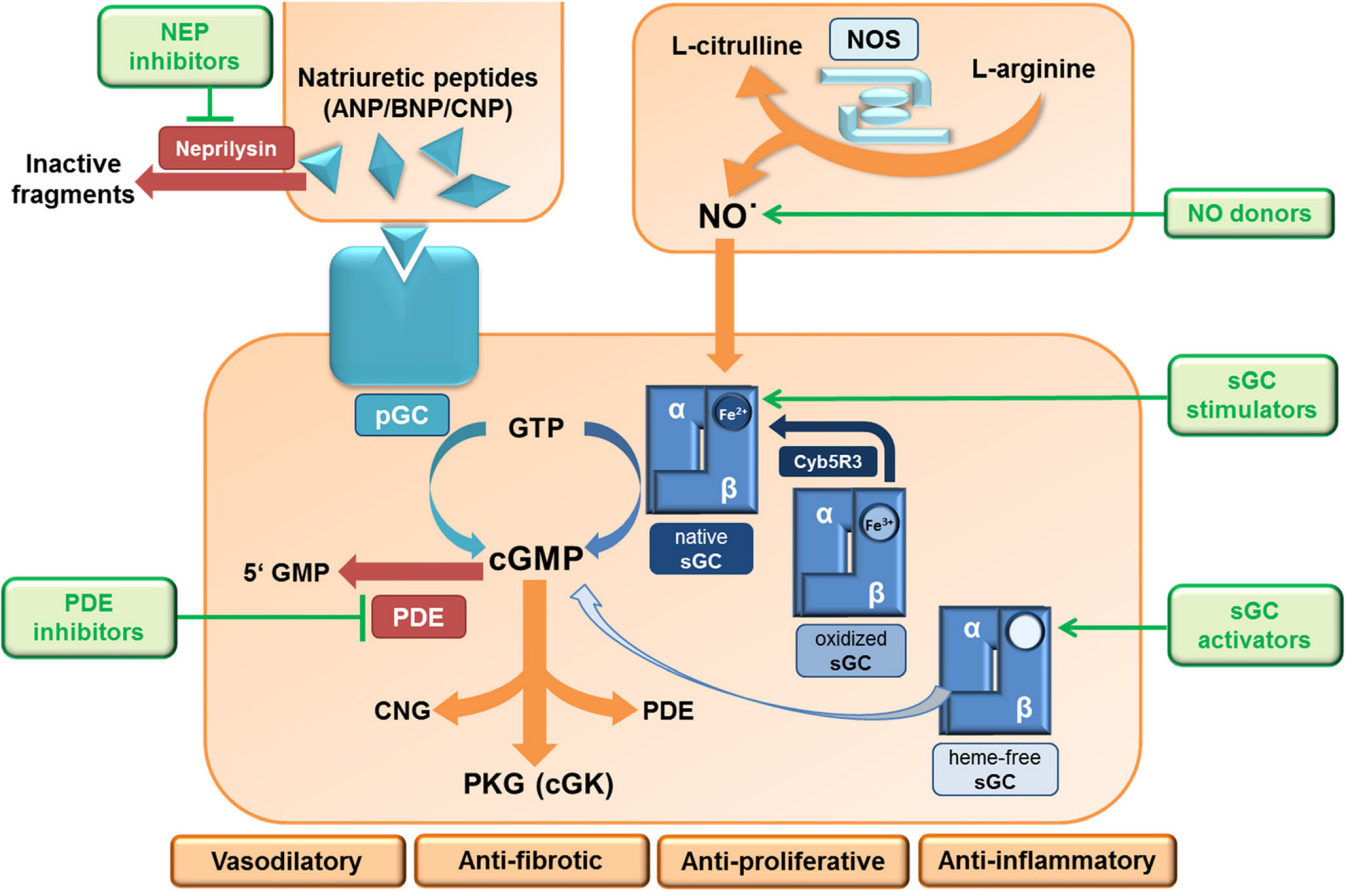
ANP, atrial natriuretic peptide; BNP, brain natriuretic peptide; cGK, cGMP-dependent protein kinase; cGMP, cyclic guanosine monophosphate; CNG, cyclic nucleotide-gated ion channels; CNP, C-type natriuretic peptide; GTP, guanosine triphosphate; GMP, guanosine monophosphate; NO, nitric oxide; NOS, nitric oxide synthase; NP, natriuretic peptide; PDE, phosphodiesterase; pGC, particulate guanylyl cyclase; PKG, protein kinase G; sGC, soluble guanylyl cyclase

Production of cGMP can be stimulated by organic nitrates and other NO donors, e.g., glyceryl trinitrate, sodium nitroprusside or molsidomine, which—enzymatically or non-enzymatically—release NO. NO donors are primarily used for the treatment or prevention of acute angina pectoris attacks in patients suffering from coronary heart disease, hypertensive crisis, and other emergencies requiring rapid relaxation of vascular smooth muscle cells (SMC). More recently, selective stimulators of sGC have been discovered. The first potent and selective sGC stimulator was riociguat which effectively enhances cGMP production; riociguat has been approved in 2013 for the treatment of pulmonary arterial hypertension (PAH) and chronic thromboembolic pulmonary hypertension (CTEPH). In addition, new sGC stimulators like vericiguat, praliciguat, and olinciguat are currently in phase 2 and 3 clinical development for kidney disease and chronic heart failure. Recently, the combination of sacubitril, an inhibitor of the neutral endopeptidase (NEP, neprilysin) which protects natriuretic peptides from degradation, with an angiotensin AT1 receptor antagonist (valsartan) has been introduced for the treatment of chronic heart failure (HF), especially in patients with reduced ejection fraction. Successful examples for targeting cGMP hydrolysis are PDE5 inhibitors. In 1999, the first selective PDE5 inhibitor, sildenafil, was introduced for the treatment of erectile dysfunction (ED) followed by vardenafil and tadalafil in 2003. Later on, sildenafil and tadalafil were also approved for the treatment of pulmonary hypertension, and tadalafil got approval for the treatment of symptoms of benign prostatic hyperplasia (BPH). Furthermore, various drugs targeting cGMP signaling are in clinical development for different diseases. The NO/sGC and NP/pGC pathways as their major pharmacological intervention sites are illustrated in Fig. 1 (published in Krishnan et al. 2018, adapted).

**Fig. 2:**
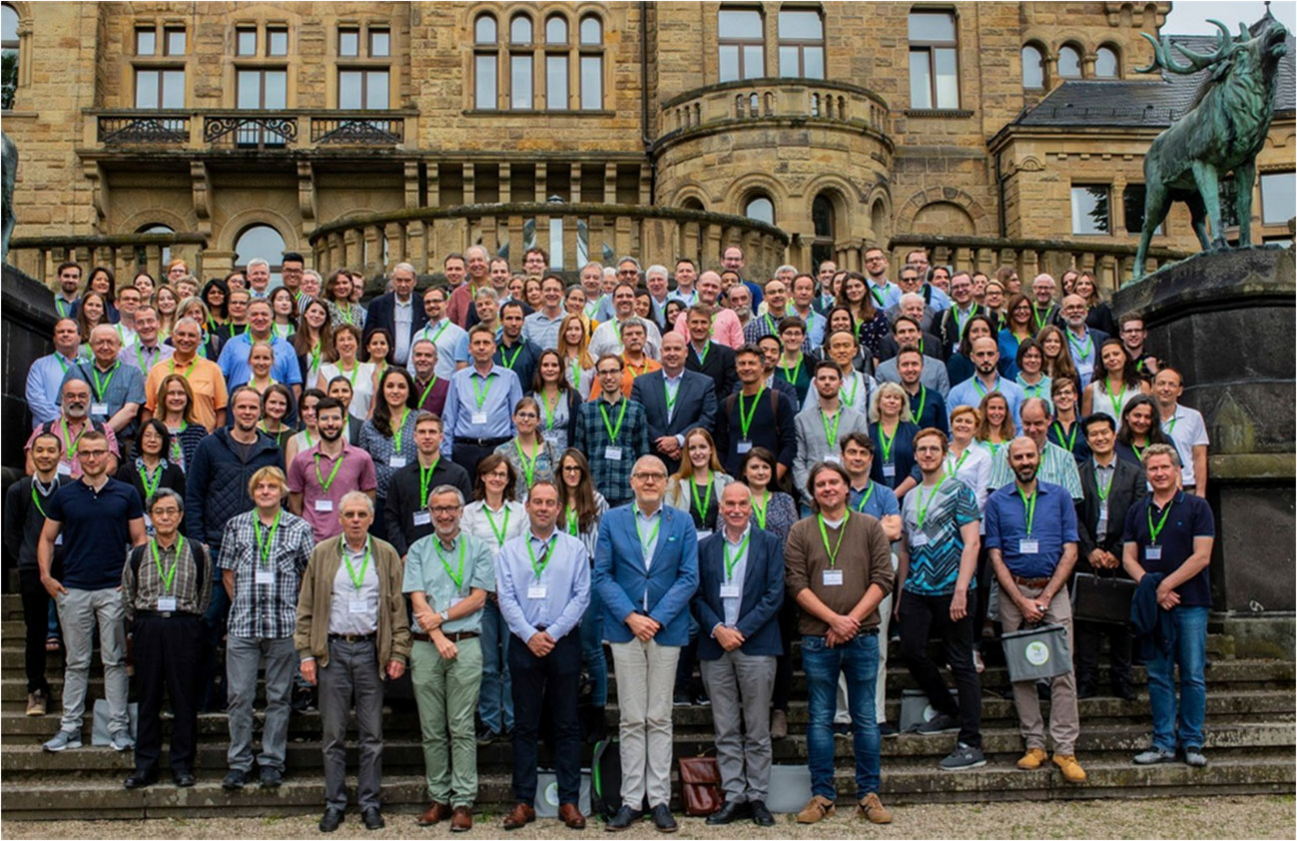
Participants of the 9th International Conference on cGMP, held in Mainz, Germany, in 2019 from June 14th–16th. For program and meeting information see https://www.cyclicgmp.net/index.html; for abstracts see 10.1186/s12967-019-1994-0 (J Transl Med 2019, 17(Suppl 2):254)

Given the broad importance of cGMP signaling, this research field ranges from cGMP generation to pharmacological interventions and from basic science to clinical applications. These efforts are reflected in a high number of publications every year. However, in addition, every second year, roughly 150 scientists from all over the world, from academia and industry including basic and translational researchers and also drug discovery experts and clinical scientists, convene for an international conference on cGMP research. The 9th meeting was held in June 2019, close to Mainz at Waldthausen (Fig. 2), including Ferid Murad, who was awarded the 1998 Nobel Prize in Physiology or Medicine for the discovery of NO. Thus, this cGMP conference is a unique opportunity to bring together all key researchers in the field. During 3 days, more than 40 talks and about 50 posters were presented and intensively discussed. In this meeting, after years of substantial efforts, a crystal structure of the full-length sGC was disclosed, definitely one of the highlights of this conference.

## New developments in cGMP signaling

### sGC structure and function

Soluble/NO-sensitive guanylyl cyclase (sGC), a heterodimeric enzyme built by two subunits α and β, is accepted as the physiological receptor for the signaling molecule NO. Depending on the α subunit, two isoenzymes are differentiated, sGC1 and sGC2 (subunit composition α_1_β_1_ and α_2_β_1_, respectively). Activation of the enzyme and thus of cGMP target proteins such as cGMP-dependent protein kinase (PKG or cGK) is pharmacologically exploited by the application of NO donors, and of sGC stimulators and activators. Many questions regarding sGC have been investigated over the last 30 years, including (1) identification and cloning of the genes for the subunits, (2) the mechanism of NO activation, (3) identification of non-NO compounds that activate the enzyme, (4) structure of the enzyme, and (5) physiological and pathophysiological functions in different species (for review see e.g. Buys et al. 2018; Childers and Garcin 2018; Friebe et al. 2018; Horst and Marletta 2018; Koesling et al. 2016). But still these and many other unsolved issues motivate researchers to produce novel and fascinating data, part of which were presented in Mainz.

Despite huge efforts over decades, many labs failed to obtain the full-length crystal structure of sGC. In this regard, the 2019 cGMP conference was outstanding regarding the latest findings on the sGC crystal structure. The GC1 from the hawkmoth *Manduca sexta* has been shown to be highly homologous to mammalian sGC1 with regard to stimulator binding. Truncation mutants (GC1-NT) lacking the cyclase domain but retaining the highly conserved coil-coil (CC) domain which links heme binding and catalytic domains were obtained in large quantities for structure/function studies. GC1-NT still binds sGC stimulators such as Bay 41-2272, a compound widely used to investigate the mechanism of sGC stimulation. Based on the structure of Bay 41-2272, a photolabile sGC stimulator, IW-854, was introduced and both stimulators share a similar sGC binding site (Wales et al. 2018). Following photoaffinity labeling with IW-854 and subsequent mass spectrometry, the binding site for stimulator compounds was identified on the β1 subunit. Additional studies were performed to find out how the gaseous activators NO and CO enhance catalysis (Weichsel et al. 2019). A stabilizing mutation within the CC domain reduces CO affinity 5-fold whereas shortened CC domains enhance CO binding. These data indicate that NO binding to the heme group induces a twist in the coiled coil. Thus, the heme binding domain communicates with the catalytic domain through the CC domain (William Montfort, Tucson).

In fact, these insights into sGC structure were extended by work from a different lab also using the *M. sexta* GC. Cryo electron microscopy was used to delineate the full-length structure of sGC. In a beautiful set of data, sGC was demonstrated to display two distinct conformations for the inactive and active state (Michael Marletta, Berkeley). Central element is the CC domain which is rigid under inactive conditions. Upon NO binding, the CC domain stretches out to induce a large conformational extension of the enzyme in order to transduce the activating signal to the catalytic domain. YC-1, and probably other stimulators bind to the top part of the CC domain to force a straightened conformation. The full array of the crystal data will definitely be a big step in order to understand sGC structure, its mechanism of activation as well as the enzyme’s binding sites for all activity-modifying compounds.

Just a few weeks after the conference, a cryo-electron microscopy structure of the human sGC α1β1 heterodimer was published in September 2019 by an independent research group, including the description of the inactive and NO-activated states at a resolution below 4 Å (Kang et al. 2019). Similar to the *Manduca sexta* enzyme, human sGC was shown to have a “bent” structure in the inactive state which stretches into a “dumbbell” shaped form after NO activation. Activation was shown to be mediated by a large overall conformational change including extension of the coiled coil domain. In the same month, the data from the Marletta group were published (Horst et al. 2019) allowing now comparison of the two crystal structures. Although the two different sets of crystal data are of course of extremely high interest, such a comparison is beyond the scope and the aim of the current manuscript. However, elucidation of the sGC structure in 2019 will remain a landmark-finding for the whole cGMP field.

### pGC structure and function

Next to sGC, the alternative cGMP source is the membrane-spanning, particulate guanylyl cyclase (pGC), which is activated by different types of peptides. Seven pGC forms have been identified in mammals: GC-A to GC-G (Potter 2011; Kuhn 2016). The best characterized pGC are GC-A (also called NPR-A or NPR1) and GC-B (NPR-B or NPR2), both receptors for natriuretic peptides. A-type or atrial natriuretic peptide (ANP) is highly expressed in the atria and released by atrial wall stretch resulting from increased intravascular volume. BNP was initially purified from brain extracts and referred to as “brain natriuretic peptide.” As subsequent studies detected much higher BNP concentrations in cardiac ventricles during cardiac stress such as congestive heart failure or myocardial infarction, it is now termed “B-type natriuretic peptide.” C-type natriuretic peptide (CNP), which does not stimulate natriuresis at physiological concentrations, is expressed in chondrocytes, endothelial cells, the heart, distinct neuronal populations, and other tissues (Potter et al. 2006). The three structurally related natriuretic peptides differ in their receptor binding as ANP and BNP both activate GC-A, whereas CNP activates GC-B (Kuhn 2016).

Recent findings about paracrine anti-inflammatory actions of CNP in the vascular system in tissue-specific knockout mice indicate that endothelial CNP, by binding to GC-B, may prevent excessive activation of certain types of immune cells during ischemia (Michaela Kuhn, Würzburg). These data suggest that CNP/GC-B/cGMP signaling contributes to the maintenance of vascular integrity and attenuates ischemic tissue damage.

### cGMP downstream targets structure and function

cGMP signaling is conveyed by several effectors including PKG, cGMP-gated ion channels and phosphodiesterases. A major cGMP effector is PKG-I, which plays a key role in the regulation of smooth vascular and cardiac function (Hofmann 2018). PKG-I is a homodimeric enzyme that is expressed as two isoforms, PKG-Iα and Iβ. After activation, PKG-I signals through phosphorylation of various proteins in a tissue-specific manner. Research over the past decade has established that PKG-I may also be activated by oxidizing agents such as hydrogen peroxide. The in vivo relevance of this cGMP-independent redox activation of PKG-I is being increasingly recognized.

The pathophysiological relevance of oxidative stress on NO/sGC signaling and the impact on disease progression is still poorly understood. However, recently and also presented on the last cGMP meeting in 2017, it was shown that vascular SMC express a reductase (CYB5R3) turning back oxidized sGC into the native form (Shah et al. 2018). More recently, it has been demonstrated that knock out of CYB5R3 in vascular SMC is linked to a hypertensive phenotype by reducing NO-mediated sGC stimulation (Durgin et al. 2019). Besides sGC, other signaling molecules in the NO and NP pathway may be very sensitive to oxidative stress, too. At the cGMP conference in Mainz, two talks focused on redox regulation of PKG-Iα. First, nitroxyl (HNO) donors, which hold therapeutic promise as vasorelaxants, were shown to induce an interdisulfide bond involving Cys42 of two PKG-Iα subunits and an intradisulfide bond between Cys117 and Cys195 in the cGMP-binding site of PKG-Iα (Friederike Cuello, Hamburg). Both disulfides were shown to enhance the kinase activity, thereby inducing vasorelaxation (Donzelli et al. 2017). Experiments in knockin mice carrying specific cysteine mutations of PKG will help to further elucidate the mechanisms underlying the effects of HNO in vivo.

The second talk investigated the molecular basis of the constitutive activation of PKG-Iα by oxidizing agents (Thomas M. Moon, Tucson). Data from an in vitro assay suggest that constitutive activation of PKG-Iα is not mediated via Cys42, but rather through oxyacid formation at Cys117. This mechanism could be particularly relevant to PKG anchored near hydrogen peroxide sources or under pathophysiological conditions associated with high intracellular concentrations of hydrogen peroxide (Sheehe et al. 2018).

Another research group recently determined the crystal structure of a PKG-Iβ holoenyzme complex at 2.3 Å that enabled to visualize the regulatory and catalytic domain interface of the inhibited state (Choel Kim, Houston). A key finding was that the regulatory domain of each monomer binds the catalytic domain of the other monomer, giving rise to inhibition in trans. This structure suggests that activated and inhibited states of PKG-Iβ are stabilized by domain–domain contacts that either expose or occlude the active site.

### cGMP PDE structure and function

The importance of cGMP for various body functions requires a tight control of its intracellular concentration. Phosphodiesterases (PDEs) are responsible for breakdown of cyclic nucleotides. In mammals, twenty-one PDE genes have been identified, which are classified into at least 11 families (PDE1 to PDE11), each with distinct substrate specificity, regulatory properties, and tissue distribution. According to preferences for cyclic nucleotides, cGMP-specific PDEs (PDE 5, 6, and 9) and cAMP-specific PDEs (PDE 4, 7, and 8) show strong specificities for either cGMP or cAMP as substrate, whereas dual-specificity PDEs (PDE 1, 2, 3, 10, and 11) may hydrolyze both cyclic nucleotides (Francis et al. 2011; Baillie et al. 2019). PDE5, PDE6, and PDE9 are of particular importance for the field since they very efficiently hydrolyze sGC- and pGC-produced cGMP (Lee et al. 2015). In addition, PDE2 and PDE3, whose activity may be modulated by cGMP, are also involved in NO and NP signaling. More recently, it was also demonstrated that PDEs are able to cleave non-canonical cNMPs (in particular, cCMP and cUMP) in addition to hydrolyzing the canonical cNMPs, cAMP, and cGMP (Schneider and Seifert 2017) which may broaden the therapeutic impact of PDE inhibitors in the future. In recent years, it turned out that PDE inhibition provides effective treatment approaches in various diseases (Baillie et al. 2019).

Whereas PDE5 inhibitors represent an approved and established treatment paradigm ranging from erectile dysfunction, pulmonary arterial hypertension to benign prostatic hyperplasia, PDE9 inhibitors are still not available for patients. In preclinical studies, PDE9 inhibitors enhanced the performance of animals in various cognition tasks in models of Alzheimer’s disease (AD), schizophrenia, and Huntington’s disease (Dorner-Ciossek et al. 2017). However, in clinical trials PF-04447943, a PDE9 inhibitor from Pfizer, had no effect on cognition in patients with mild to moderate AD. Boehringer Ingelheim also investigated their PDE9 inhibitor BI 409306 as a cognition enhancer in phase 2 clinical trials for both AD and schizophrenia (NCT02337907 and NCT02281773; Frölich et al. 2019; Brown et al. 2019). In 2018, Boehringer Ingelheim announced that efficacy endpoints in AD were not met and that beneficial effects in schizophrenia were also not found. These data question the concept of PDE9 inhibition for dementia and a careful analysis of the research results is needed.

In line with these data, new findings about PDE9A signaling in the brain were presented (Frank Menniti, Kingston). Pharmacological inhibition of PDE9A with a specific inhibitor resulted in increased cGMP levels in cortex, hippocampus, and striatum. However, this PDE9A-regulated active cGMP pool was not altered by genetic deletion of NO synthases, PDE1B and PDE10A, nor by pharmacological modulation of dopamine and glutamate signaling. These data suggest that PDE9A regulates NO-independent cGMP signaling in the brain. They further point to a compartmentalized cGMP pool that is independent from PDE1B and PDE10A (Harms et al. 2019). Further information on the interplay between different PDEs may be of importance for future CNS programs in the field of cognition enhancement.

Recent studies showed that PDE10A expression is upregulated in certain cancer models suggesting that PDE10A-mediated signaling is involved in tumor cell growth (Zhu et al. 2017). New PDE10A-inhibiting lead compounds that do not cross the blood brain barrier are being developed for cancer treatment (Gary Piazza, South Alabama). In mouse models, high concentrations of one of these compounds were detected in colonic mucosa, while another compound was enriched in lung tissue. Their anticancer activity in models of colon and lung cancer indicated that PDE10 may be a novel target for cancer therapy. However, it remains to be elucidated whether the beneficial effects of inhibition of this dual-specificity PDE are mediated by cGMP and/or cAMP.

To further elucidate the impact of PDEs on canonical and non-canonical cNMPs, it is important to precisely quantify cNMPs in biological samples. In this respect, a highly sensitive and high-throughput mass spectroscopy method was introduced and offered to the research community (Roland Seifert, Hannover) with which cNMPs could be quantified in cells, tissues, blood, and urine (Bähre et al. 2014, 2015).

## New developments in cGMP-based treatment approaches and therapeutic applications

### The role of cGMP in cardiovascular diseases, heart failure, and chronic kidney disease

Nitric oxide and natriuretic peptides play a pivotal role in regulation of the cardiovascular system and are critically involved in heart disease and heart failure (Farah et al. 2018). LCZ696, a combination of the neprilysin inhibitor sacubitril (Sac) with the angiotensin receptor blocker valsartan (Val), has been approved as a novel therapeutic strategy in chronic heart failure with reduced ejection fraction (HFrEF) in 2015. Sac inhibits natriuretic peptide degradation and, thereby, elevates cGMP levels. In the phase 3 study PARADIGM-HF, LCZ696 significantly reduced cardiovascular mortality and reduced the risk of cardiovascular death and hospitalizations related to heart failure versus the ACE inhibitor enalapril. Interestingly, LCZ696 very recently failed the primary endpoint in a phase 3 clinical trial in HFpEF (PARAGON-study, Solomon et al. 2019). In addition to LCZ 696, two sGC stimulators are currently being developed for the treatment of heart failure, namely vericiguat and praliciguat. After overall encouraging results in phase 2 clinical trials with the sGC stimulator vericiguat (Gheorghiade et al. 2015; Pieske et al. 2017; Filippatos et al. 2017), vericiguat is currently in phase 3 clinical development for the treatment of HFrEF (Armstrong et al. 2018). In addition, the sGC stimulators vericiguat and praliciguat are in phase 2 clinical development for the treatment of heart failure with preserved ejection fraction (HFpEF) (Butler et al. 2019). These studies have recently been completed and results are expected for the end of 2019 or beginning of 2020. These data will provide valuable insights in the treatment potential of sGC stimulation in HFrEF and HFpEF (Table 1).

**Table 1:** Phase 2/3 clinical trials with pGC- and sGC-modulating drugs in cardiovascular diseases and chronic heart failure

ME	Phase	Indication	NCT number	Study name	Status	Reference
Sac/Val; LCZ696	2	HFpEF	NCT00887588	PARAMOUNT	Completed	Solomon et al. 2012
Sac/Val; LCZ696	2	HFrEF	NCT01922089	TITRATION	Completed	Senni et al. 2016
Sac/Val; LCZ696	3	HFrEF	NCT01035255	PARADIGM-HF	Completed/approved	McMurray et al. 2014
Sac/Val; LCZ696	3	HFpEF	NCT01920711	PARAGON-HF	Completed	Solomon et al. 2019
Riociguat	2	PH-LVD	NCT01065454	LEPHT	Completed	Bonderman et al. 2013
Vericiguat	2	HFrEF	NCT01951625	SOCRATES-Reduced	Completed	Gheorghiade et al. 2015
Vericiguat	2	HFpEF	NCT01951638	SOCRATES-Preserved	Completed	Pieske et al. 2017
Vericiguat	3	HFrEF^a^	NCT02861534	VICTORIA-HFrEF	Ongoing	Armstrong et al. 2018
Vericiguat	3	HFpEF^a^	NCT03547583	VITALITY-HFpEF	Ongoing	Butler et al. 2019
Praliciguat	2	T2D and HTN	NCT03091920		Completed	
Praliciguat	2	T2D and HTN	NCT02906579		Completed	
Praliciguat	2	HFpEF	NCT03254485	CAPACITY-HFpEF	Ongoing	
Praliciguat	2	Diabetic nephrop.	NCT03217591		Ongoing	

^a^Bayer AG/MSD codevelopment of vericiguat

Despite the drug approval of LCZ696 and the late stage clinical developments with sGC stimulators, the molecular mode of action, how cGMP increase exerts protective effects in cardiovascular and heart diseases is only understood in part. This is important since the clinical effects with these drugs need to be further explored in order to define the most appropriate patient population. Moreover, this also could help to understand the failure of cGMP-modulating drugs, e.g., in the HFpEF patient cohort. Our knowledge gaps start already with the cellular und subcellular distribution of sGC and pGC, including the expression of cGMP-degrading PDEs in the cardiovascular system. However, on a functional level, it has been shown that cGMP could influence cardiac contractility, cardiac remodeling, and cardiac hypertrophy. Since cardiovascular diseases and heart failure are also driven by comorbidities which affect not only the heart but also blood vessel function and stiffness or renal and adipose tissues, the focus needs to be broader. Other mechanisms, such as vascular permeability, sympathetic drive, glucose tolerance, lipid metabolism, or effects on platelets, need to be integrated and better understood. This will also help to put these potential other mechanisms in perspective to the well-described relaxation of SMC, leading to blood pressure and afterload reduction. Finally, cyclic nucleotide signaling seems to be highly orchestrated and compartmentalized in cells, and crosstalk between cGMP and cAMP, and also between sGC and pGC obviously plays a role, too. This call for a better understanding of all these effects is reflected in the vast literature of the recent years and also in the research presented at the cGMP conference.

The function of sGC in cardiomyocytes has been a matter of debate during the last years. Some of the discussed issues are (1) sGC expression is very low; (2) as a consequence, NO-induced cGMP increases have been barely measurable (Götz et al. 2014); (3) NO released from either myocytes or endothelial cells is thought to be very effectively scavenged by myoglobin in cardiac myocytes (Wykes and Garthwaite 2004). However, cardiomyocyte-specific deletion of sGC in mice had a negative effect on cardioprotective signaling following acute myocardial infarction in vivo (Frankenreiter et al. 2018). Intriguingly cardiac fibroblasts/fibroblast-like cells can serve as major source of cGMP for cardiomyocytes (Menges et al. 2019). Isolated murine cardiomyocytes expressing the Förster resonance energy transfer (FRET) indicator cGi-500 (EC_50_ 500 nM) were neither responsive to NO donors at high concentrations nor did sGC activators or stimulators in the presence of IBMX induce changes in cGMP measured by using the FRET technique (Doris Koesling, Bochum). In contrast, cardiac fibroblasts (or a fibroblast-like cell type) produced enormous cGMP FRET signals when challenged with aforementioned compounds. In fact, cGMP signals were found to be as high as in SMC. This property appears to be specific for cardiac fibroblasts as fibroblasts of dermal origin only showed 20-fold lower cGMP response to NO. When co-culturing fibroblasts and cardiomyocytes, in which only the latter carried the cGMP FRET sensor, sGC stimulation now led to robust cGMP increases in cardiomyocytes. This response was inhibited by a series of different gap junction blockers such as carbenoxolone or GAP26 indicating gap junctions to shuttle cGMP from fibroblast into cardiomyocytes. Accepting this cGMP crosstalk between different cardiac cell populations, it is of course mandatory to be proven in intact heart. How fibroblast sGC is regulated, how it affects cardiomyocyte function, and whether gap junction transport is also regulated by cellular factors, are only few questions that arise from these data.

In line with the clinical experiences, a preclinical study showed that the Sac/Val combination improved left ventricular function in a mouse model with chronic pressure overload caused by transverse aortic constriction (TAC) (Robert M. Blanton, Boston). In the TAC wildtype mice, both Val and Sac/Val reduced left ventricular (LV) hypertrophy. However, in TAC mice with a mutated form of PKG-Iα, the Sac/Val combination improved LV fractional shortening and LV systolic function, too, whereas Val alone had no effect when compared with vehicle. These data are suggesting a beneficial effect of the Sac/Val combination which not or not fully be mediated by the classical PKG-I pathway (Tam et al. 2019).

To study the potential mechanism and beneficial effects of ANP and BNP through GC-A receptor stimulation in the heart, mice expressing a mutant GC-A that mimics a constitutively phosphorylated enzyme, were generated (Jerid Robinson, Minneapolis). The exchange of 8 conserved phosphorylation sites to glutamate leads to a 2–4-fold increase in ANP-dependent guanylyl cyclase activity. Although the blood pressure was not significantly different in mice with higher GC-A activity compared with WT mice, activation of ANP/BNP signaling resulted in decreased heart size as well as increased adiposity. These effects were seen in male mice only and were paralleled by elevated testosterone levels. The role of increased testosterone levels to the heart and adipose effects is currently under investigation. However, these data could also suggest that ANP and BNP may have sex-specific effects on heart hypertrophy and adipose tissue (Robinson et al. 2019).

Besides ANP and BNP signaling, there is currently also a focus on the GC-B ligand CNP. To this end, the effects of CNP were investigated in an experimental model of chronic HF induced by 6-week coronary artery ligation in rats (Lise R. Moltzau, Oslo). CNP, by stimulating GC-B, was able to enhance β1-adrenoreceptor- and 5-HT_4_-mediated inotropic responses in left ventricular strips from sham-operated and HF mice in vitro. These responses could be amplified by PDE2 inhibition in sham-operated but not HF mice indicating a different compartmentalization status in healthy versus diseased mice. Taken together, these data suggest a beneficial role of pGC stimulation by either natriuretic peptides or inhibitors of natriuretic peptide degradation. However, the subcellular distribution of these signaling cascades in microdomains which may account for the different effects is not clear yet.

In order to better understand these microdomains and compartmentalization of cGMP signaling, especially in the heart, targeted FRET-based biosensors expressed in transgenic mice were employed in order to monitor cytosolic and microdomain-specific cGMP production (Viacheslav Nikolaev, Hamburg). Whereas GC-B seems uniformly localized on the cardiomyocyte membrane, functional GC-A receptors are found exclusively in transverse (T)-tubules. Although both ANP and CNP protect from cardiac hypertrophy, their effects on contractility are markedly different, from almost no effect (ANP) to strong negative inotropic and positive lusitropic responses (CNP) with the underlying mechanisms being still unclear. The CNP membrane localization is associated with CNP/GC-B/cGMP signals which diffuse over long distances inside the cell, whereas ANP/GC-A/cGMP signals are highly confined to T-tubular microdomains by local pools of PDE2 activity. Although these results need further investigation, they may explain the different functional effects of ANP and CNP signaling in the heart (Nikolaev 2019).

In addition, new FRET-based biosensors for detection of cGMP were presented (Kjetil W. Andressen, Oslo). These biosensors (termed *Pf*PKG) were produced by flanking the cyclic nucleotide binding domain of *Plasmodium falciparum* cGKI with different FRET pairs. Experiments in rat cardiomyocytes and stellate ganglion neurons revealed that the *Pf*PKG biosensors display high affinity for cGMP (Calamera et al., 2019). Hence, they are likely to serve as new tools for real-time measurement of low intracellular cGMP concentrations in living cells.

Since cGMP signaling is significantly regulated by cGMP degradation, intense investigations of PDE distribution, function, and regulation in the cardiovascular system have been performed. In clinical trials, PDE5 inhibitors improved patient exercise capacity and ventricular function in HFrEF and showed a small to moderate benefit on the composite endpoint of death and hospitalization (Vecchis et al. 2017), but were ineffective in HFpEF (Redfield et al. 2013). Although clinical trials with PDE5 inhibitors in HFpEF were not successful, it is important to understand which PDEs are involved in cardiovascular function and heart failure. To this end, in the “F. Murad Lecture,” David A. Kass (Baltimore) summarized his long-lasting experience on PDE research in the cardiovascular system with a focus on the signaling on potential role in compartmentalization and crosstalk.

cGMP is also involved in the regulation of cardiac sympathetic drive (Li and Paterson 2019). Reduced cGMP levels, due to impaired NO and natriuretic peptide signaling, have been shown to disrupt intracellular calcium homeostasis in sympathetic neurons and to increase cardiac sympathetic neurotransmission. A recent study revealed that PDE2A expression is upregulated in stellate ganglia from patients and rats with sympathetic hyperactivity. Notably, inhibition or downregulation of PDE2A improved calcium balance and neurotransmission. These findings suggest that the sympathetic hyperactivity during diseases such as hypertension and heart failure is associated with dysregulation of PDE2A-controlled cyclic nucleotide signaling. Thus, site-specific targeting PDE2A may provide a new therapeutic strategy (David J. Paterson, Oxford).

Another study focused on cardiovascular PDE2A signaling. In a mouse model of heart failure, inhibition of PDE2A increased cGMP levels in whole heart homogenates and reversed various pathological indices such as left ventricular hypertrophy, cardiac fibrosis, and impaired contractility (Michael E. J. Preedy, London). Interestingly, the beneficial effects of PDE2A inhibition were absent in mice lacking the α1 subunit of sGC, further supporting the finding that PDE2A controls NO/sGC/cGMP signaling during heart failure (Baliga et al. 2018).

The effects of sGC stimulation on the cardiovascular and cardiorenal systems are probably very complex based on to the broad distribution of this pathway in different cell types and tissues. Besides the well-established hemodynamic effects of sGC stimulators, mainly driven by vascular smooth muscle relaxation, additional effects on blood glucose levels and lipids may exist. In a high-caloric diet-induced obesity mouse model, the sGC stimulator praliciguat was shown to reduce triglyceride levels and increase energy expenditure. In addition, an improvement of insulin sensitivity could be demonstrated (Juli Jones, Boston). These results could point towards a new mode of action of sGC stimulators important in cardiovascular diseases with an obese and diabetic background.

How cGMP impacts on the metabolic profile and on triglycerides is, however, not understood very well on a mechanistic level. Therefore, starting point for another study was the role of cGMP in brown adipose tissue (BAT) lipid storage (Hoffmann et al. 2015). Previous data suggested that the small GTPase Rac regulates sGCβ1 promotor activity in BAT in a VASP-dependent manner (Jennissen et al. 2012). To investigate a possible role of RAC activity in the vasculature, cGMP and Rac signaling were investigated in SMC from WT and VASP-KO mice (Alexander Pfeifer, Bonn). Surprisingly, vascular cGMP signaling was found normal in VASP-KO mice. SMC-specific overexpression of Rac failed to affect NO-induced vasorelaxation and did not affect sGCβ1 expression. In contrast to these data from murine cells/tissues, Rac overexpression in human VSMC led to reduction of the sGCβ1 subunit expression (protein and mRNA). Although still speculative, Notch, whose expression paralleled that of sGCβ1, appears to play a role in Rac signaling in human VSMC.

Expression and function of sGC in erythrocytes are still not entirely clear even though cGMP modulation in red blood cells (RBC) is conceivable to have beneficial effects on cardiovascular and embolic diseases. Several studies argue for sGC to regulate erythrocyte properties (Tsuda et al. 2000; Bor-Kucukatay et al. 2003) whereas others have provided evidence against sGC expression in these cells (Gambaryan et al. 2016; Angermeier et al. 2016). Clinically, these functions may be relevant in patients with sickle cell anemia (Kato 2015). Using a meticulous purification scheme, sGC was shown to be expressed in RBC and activation of the enzyme produced functional cGMP signaling via PKG and VASP phosphorylation (Miriam Cortese Krott, Düsseldorf). In fact, stimulation with high NO concentrations in combination with Bay 41-2272 led to 1000-fold increased cGMP levels which is surprising given the scavenging properties of hemoglobin. Lack of NO response in sGC1-KO RBC and preservation in sGC2-KO RBC indicate sGC1, but not sGC2 to be present in murine erythrocytes. Patients with stable coronary artery disease were shown to have an increased fraction of oxidized sGC based on the lack of effect of Bay 60-2270, even though sGC activity in RBC was fully preserved. Mice with a RBC-specific deletion of sGC1 α subunit showed a very strong phenotype with reduction in hematocrit and RBC numbers, in addition to splenomegaly and increased systolic and mean arterial blood pressures. How the absence of sGC in RBC induces such a strong phenotype is under intensive investigation.

Platelets have been known for a long time to express high amounts of sGC and the antithrombotic effects of NO/cGMP signaling in these cells have been extensively studied (Makhoul et al. 2018). The sGC in platelets as well as bone marrow (BM) was suggested to have protective effect in mice after cardiac arrest (Fumito Ichinose, Boston). Mice were subjected to potassium chloride-induced cardiac arrest. During/after resuscitation, the animals either breathed normal air or an NO/air mixture. NO inhalation increased survival in WT mice but failed to do so in mice lacking the α1 subunit of sGC. A role of BM sGC was shown by transplantation experiments: WT animals receiving BM from WT exhibited better survival and neurological function after resuscitation than WT mice receiving BM from sGCα1-KO animals indicating a role of BM-derived cells. In addition, platelet-specific sGCα1-deficiency also reduced survival after cardiac arrest. In summary, sGC in platelets/BM-derived cells is thought to reduce platelet activation, RBC entrapment, inflammation, and polymorphonuclear cell number in the cerebral circulation that increase in the course of cardiac arrest and resuscitation in mice.

The role of natriuretic peptide receptors in pulmonary vascular endothelial function is another highly interesting field of investigation. Previous studies indicate that ANP may protect against acute lung injury, but the underlying signaling mechanism remained poorly understood. In a model of acute lung injury induced by aspiration of *Pseudomonas aeruginosa*, the ability of ANP to mitigate acute lung injury was further investigated (James R. Klinger, Providence). The observations that signs of injury were altered in GC-A knockout mice and that lentiviral overexpression of GC-A enhanced the protective effect of ANP suggest that GC-A might represent a novel target for treatment of acute lung injury. More recently, it could also be shown that LCZ696 reduces pulmonary pressure in a rat model of PH and may be appropriate for treatment of pulmonary hypertension and RV dysfunction (Clements et al. 2019).

In recent years, genetic sequencing and genome-wide association studies provided further evidence that mutations in genes of the cGMP cascades are linked to cardiovascular, cardiopulmonary, and cardiorenal diseases (Wobst et al. 2018). Recently, a clinically relevant mutation of PKG-I (a missense variant of PKG-Iα with replacement of valine by isoleucine (V219I)) was detected in a patient with congenital heart disease, aortic root dilation, and aneurism formation. In various in vitro experiments, this V219I mutation was associated with a higher cGMP sensitivity of PKG-Iα (Philipp Henning, Kassel). Considering the important function of PKG-Iα in the regulation of vascular tone, the resulting increased basal kinase activity may contribute to the human disease phenotype.

News about the functions of PDE10A in the cardiovascular system were also provided during the cGMP conference (Chen Yan, Rochester). Previous genome-wide association studies in inbred mouse strains linked PDE10A to vascular occlusive disorders. Studies in primary cultured SMC, human saphenous vein explants, and mouse models revealed that PDE10A expression was upregulated after vascular injury, and that injury-induced neointimal formation was attenuated by PDE10A knockout or inhibition. Interestingly, PDE10A seems to regulate SMC proliferation by modulating cGMP signaling. These results point to PDE10A as a new potential target for proliferative vascular disorders.

However, it became very obvious at the cGMP conference that there are still controversies and significant knowledge gaps regarding the role of cGMP in cardiovascular, cardiopulmonary, and cardiorenal diseases, as in heart failure and kidney disease. The cellular localization and expression of sGC, pGC, PKG, and PDEs, the potential species differences of cGMP signaling, and also the therapeutic impact of sGC and pGC stimulation also in relationship to common comorbidities, still need further clarification. This understanding will help to develop more personalized cGMP therapies.

### The role of cGMP in fibrotic remodeling and fibrotic diseases

Fibrotic remodeling and fibrosis participate in various diseases and also seem to be associated with aging. Increasing evidence over the last years indicates that sGC plays a regulatory role in wound healing and fibrotic responses (Sandner and Stasch 2017). Although cumulative evidence suggested an antifibrotic effect of cGMP, the majority of these studies were descriptive and molecular insights and mechanistic studies are missing, even expression data on the signaling pathways in fibroblasts, myofibroblasts, or pericytes are not systematically available.

Pericytes are key cells for fibrosis and pericytic sGC expression has been shown in all organs so far investigated (Friebe et al. 2018). Fibrotic responses in lung, liver, and skin are likely to involve sGC and the formation of myofibroblasts. In all three organs, sGC was shown to be expressed in pericytes and also in SMC (Dieter Groneberg, Würzburg). Lineage tracing using the Ai14 tdTomato reporter helped to identify pericytes and hepatic stellate cells—the respective counterpart in liver—as progenitors of myofibroblasts which develop in the course of a wound healing response. The identification of sGC in skin pericytes is intriguing but its role during wound healing and scar formation in skin has yet to be shown.

### The role of cGMP for neuronal function and the treatment of CNS diseases

The development of modulators of cGMP signaling for use in central nervous system (CNS) diseases has lagged behind their application in cardiovascular or other peripheral disorders. This is surprising based on the central role of cGMP in learning and memory, and the substantial evidence that cGMP signaling is perturbed in CNS disorders including Alzheimer dementia (AD), Huntington disease, and schizophrenia. However, several development programs targeting cGMP signaling in the CNS have been started (Hollas et al. 2019; Baillie et al. 2019). The fact that initial results with PDE5 and PDE9 inhibitors have failed (as reported before) could be related to low cGMP production in CNS diseases or to involvement of other PDEs in cGMP degradation in the CNS. (Heckman et al. 2019).

Preclinical studies with a CNS-penetrant sGC stimulator (IW-6463) which stimulates cGMP production in the brain and CNS were also reported, evaluating its potential for treatment of serious CNS diseases (Christopher Winrow, Boston). In rodents, this compound improved cerebral blood flow, alleviated LPS-induced neuroinflammation, provided neuroprotective effects measured by synaptic spine density, and improved neuronal function in behavioral assays. A clinical phase 1 trial analyzing safety, tolerability, pharmacokinetics, and pharmacodynamics of IW-6463 in healthy subjects has been started whose topline data are expected at the end of 2019.

The potential of inhaled NO for therapy of neurological disorders was also addressed. Previous studies had revealed that reduced endothelial NO production in the cerebral vasculature is often associated with neurological disorders such as ischemic and hemorrhagic stroke and traumatic brain injury. The reduced NO production is supposed to further damage the brain and to increase the loss of neuronal function. New preclinical data suggest that NO is preferentially released in vascular territories with reduced oxygen partial pressure and that brain damage after acute injury may be reduced by inhaled NO. A first clinical trial testing the effects of inhaled NO in moribund patients is ongoing (Nikolaus Plesnila, München).

Recent data indicate a role of pGC signaling in auditory function (Wolter et al. 2018). Tissue staining experiments showed that ANP, BNP, and GC-A are expressed in distinct cellular populations of the hearing organ. Preclinical studies analyzing the hearing function of GC-A knockout mice suggest that GC-A signaling is involved in age-related hearing impairment and noise-induced hearing loss. Hence, GC-A could be a new target for improvement of auditory function (Lucas Ruettiger, Tuebingen).

### The role of cGMP for the treatment of rare diseases

In addition to the broad cardiovascular indications, it became obvious in recent years that cGMP-enhancing therapies offer new treatment options in difficult-to-treat and rare diseases. This was underlined by the first-in-class approval of the sGC stimulator riociguat (BAY 63-2521) for the treatment of two rare forms of pulmonary hypertension, pulmonary arterial hypertension (PAH), and chronic thromboembolic pulmonary hypertension (CTEPH). In addition, it has been shown that cGMP elevation by sGC stimulators and sGC activators may have therapeutic benefits in sickle cell disease (SCD), systemic sclerosis (SSc), and muscular dystrophies such as Duchenne muscular dystrophy (DMD). Although clinical data are still—at least in part—missing, preclinical findings provide steadily growing and supportive evidence (Table 2).

**Table 2:** Phase 2/3 clinical trials with sGC drugs in rare diseases

ME	Phase	Indication	NCT number	Study name	Status	Reference
Riociguat	3	PAH	NCT00810693	PATENT	Completed/approved	Ghofrani et al. 2013a
Riociguat	3	CTEPH	NCT00855465	CHEST	Completed/approved	Ghofrani et al. 2013b
Riociguat	3	PAH	NCT00863681	PATENT-2	Completed/approved	Rubin et al. 2015
Riociguat	3	CTEPH	NCT00910429	CHEST-2	Completed/approved	Simonneau et al. 2016
Riociguat	3	pediatric PAH	NCT02562235	PATENT-CHILD	Ongoing	
Riociguat	3	PAH	NCT02007629	RESPITE	Completed	Hoeper et al. 2017
Riociguat	2	IIP	NCT02138825	RISE-IIP	Terminated	Nathan et al. 2019
Riociguat	2	SSc	NCT02283762	RISE-SSc	Completed	Distler et al. 2017
Riociguat	2	SCD	NCT02633397	STERIO-SCD	Ongoing	
Olinciguat	2	SCD	NCT03285178	STRONG-SCD	Ongoing	
Olinciguat	2	Achalasia	NCT02931565		Completed	

#### Sickle cell diseases

The role of NO and cGMP for the pathology of SCD has been recently reviewed (Kato 2015; Kato et al. 2017; Conran and Torres 2019). In SCD, hemolysis releases cell-free hemoglobin into the plasma, which can scavenge NO and generate reactive oxygen species impairing redox balance and leading to proliferative systemic and pulmonary vasculopathy. Since this is one of the key pathological mechanisms in SCD, NO-independent stimulation has shown beneficial effects in preclinical models and currently two sGC stimulators, riociguat and olinciguat, are in phase 2 clinical development in SCD patients (Mark Gladwin, Pittsburgh). Beyond sGC stimulators, PDE9 inhibitors have been profiled in preclinical and early clinical studies for treatment of SCD in recent years (Conran and Torres 2019). PDE9 is expressed in hematopoietic cells, with even higher expression in the neutrophils and reticulocytes of patients with SCD (Almeida et al. 2008). The selective PDE9 inhibitor BAY 73-6691 demonstrated preclinical efficacy in vitro and in vivo SCD models (Almeida et al. 2012). A very recent clinical phase 1 trial with the PDE9 inhibitor PF-04447943 suggested that this compound has beneficial effects by reducing vaso-occlusion in patients with SCD (Charnigo et al. 2019). In addition, the PDE9 inhibitor IMR-687 (McArthur et al. 2016) was granted rare pediatric disease designation by the FDA and is currently being evaluated in a randomized, placebo-controlled, multicenter study phase 2 trial (NCT03401112) to determine safety, pharmacokinetics, and preliminary pharmacodynamics in patients with SCD.

#### Systemic sclerosis

In recent years, the antifibrotic effects of cGMP have been intensively studied (Higuchi et al. 2015; Sandner et al. 2017; Matei et al. 2018). At the 2017 cGMP conference, the preclinical effects of sGC stimulation and riociguat on skin fibrosis were presented (Sandner et al. 2017; Friebe et al. 2017). At the present cGMP conference, the results of a phase 2 clinical program assessing the treatment effects of riociguat in SSc patients with diffuse systemic sclerosis were presented (Melanie Hemmrich, Wuppertal). Although the study did not meet its primary endpoint, fewer patients in the riociguat group showed progression of modified Rodnan skin score (mRSS) compared with placebo. There was a reduction in mRSS with riociguat vs placebo (− 2.34; 95% CI − 4.99 to 0.30; *p* = 0.08). Of note, in an exploratory analysis of subgroups, riociguat may be associated with preservation of lung function in patients with diffuse cutaneous SSc and evidence of interstitial lung disease. Riociguat also showed benefits on disease progression, digital ulcers, and Raynaud’s phenomenon with no new safety concerns identified in this patient population. These data suggest that sGC stimulation prevented the progression of fibrosis as also implied by the preclinical data. However, further clinical studies have to identify the responding subpopulations.

#### Duchenne muscular dystrophy

DMD is based on mutations within the dystrophin gene. DMD symptoms affect multiple muscular tissues and include progressive muscle loss, exaggerated post-exercise fatigue, respiratory muscle weakness, and cardiomyopathy. These skeletal and cardiac muscle dysfunctions are associated with loss of nNOS (Balke et al. 2019). Restoration of NO and thus cGMP signaling are therefore thought to help in the therapy of DMD (Moon et al. 2017). Based on the lack of nNOS-targeted approaches, a preclinical study investigated whether sGC agonists affect skeletal and myocardial symptoms in mdx mice, a mouse model for DMD (Justin Percival, Miami). In fact, sGC stimulation increased sGC expression and activity in mdx skeletal muscles. This effect was paralleled by increased cGMP levels as well as by reduced contraction-induced muscle damage and post-exercise fatigue. In hearts from aged mdx mice, sGC stimulation (BAY 41-8543) decreased hypertrophic left ventricular (LV) remodeling, diastolic function, and fibrosis. Breathing quality was increased due to diaphragm myocyte growth, improved diaphragm function, and reduced muscle weakness. Although the effectiveness of increasing cGMP signaling in defective muscle in humans is still to be shown, sGC stimulation may at least turn out as adjunct therapy to possible genetic approaches. In addition, in the poster presentation, preclinical data of the effects in mdx mice with the sGC stimulator BAY 41-2272 were shown. Although the genetic mouse model used was different (mdx/mTRG2), BAY 41-2272 improved grip strength and diaphragm pathology. However, these mice did neither exhibit a cardiac phenotype nor develop cardiac dysfunction at 36 weeks of age as demonstrated by echocardiography and invasive hemodynamics (Shalini M. Krishnan, Wuppertal). Since sGC stimulators are in development for heart failure, it will become interesting to see whether sGC stimulation could also be beneficial in DMD-caused cardiomyopathies.

### The role of cGMP for infectious diseases

Malaria remains a serious health threat in developing countries with more than 500,000 deaths and millions of infections per year (Ashley et al. 2018). Drugs against the transmitting parasite, *Plasmodium*, a protozoan parasite, have been known for a long time, but treatment increasingly fails due to resistance development. Artemisinin-based chemotherapeutics have been the latest therapeutically effective options against *P. falciparum*, but within the last 10 years, resistance has developed in Southeast Asia and is feared to import to Africa. Hence, identification of novel target structures in the parasite is mandatory for the development of alternative anti-malaria agents (Moxon et al. 2019; Narula et al. 2019).

Cyclic nucleotides have been appreciated as intracellular messengers not only in most animal cells but also in malaria parasites in which they control many stages of their life cycle (Baker et al. 2017a, 2017b). *Plasmodium falciparum* PKG (*Pf*PKG) has been identified as a promising target for antimalarial treatment. *Pf*PKG shares several properties with mammalian PKG, such as the regulation by allosteric and cooperative binding of cGMP to cyclic nucleotide binding sites (CNB) but differs in the overall domain organization. In fact, one of the two additional CNBs, CNB-D was recently shown to have a critical role in cGMP binding and enzyme activation (Kim et al. 2015).

A search for a *Pf*PKG inhibitor started with approximately 500 analogs sharing an imidazopyridine scaffold (Baker et al. 2017a, 2017b). One of them, ML10, has been identified as potent inhibitor of *Pf*PKG (David Baker, London). By the inhibition of kinase activity, ML10 inhibits merozoite egress from erythrocytes and reduces RBC invasion as well as ookinete motility indicating *Pf*PKG to be essential at all key stages. Phosphoproteome analysis revealed over 100 phosphorylation sites on 69 proteins which included PDEβ, ACβ, and GCα. ML10, although at relatively high dose, was also shown to clear blood stage infection in a mouse model engrafted with human RBC. In addition, high-throughput screening helped to identify compounds with a thiazole structure that exhibited fast speed killing properties; consequently, dual-specificity compounds may turn out to increase therapeutic effectiveness and allow for the reduction of dose.

Structural information on *Pf*PKG and its activation mechanism were also presented at the meeting. The importance of CNB-D in the activation mechanism of *Pf*PKG had already been shown (Franz et al. 2018). Expression of several deletion constructs of the enzyme which lacked either the autoinhibitory sequence (AIS) or the CNB revealed that the AIS is not needed for activation (Friedrich W. Herberg, Kassel). Of the four CNB, CNB-D, which has the highest cGMP affinity, appears mandatory for activation. A construct lacking the AIS and all CNB except CNB-D was shown to be catalytically active in the absence of cGMP and to be efficiently activated by cGMP. Consequently, deletion of CNB-D destroyed the activation mechanism. Further data showed that CNB-A and -B modulated kinase activity whereas CNB-C had no obvious function. Additional crystallographic data confirming these results and revealing further information on the regulation of PKG activation by AIS and the different CBDs have just been published (El Bakkouri et al. 2019).

Another protozoan parasite, *Toxoplasma gondii*, uses cats as intermediate hosts for sexual reproduction even though it is capable of infecting virtually all warm-blooded animals including humans resulting in toxoplasmosis. Egression of Toxoplasma parasites from infected cells is also dependent on cGMP and PKG (Dominique Soldati-Favre, Geneva). Phosphatidic acid acts as key mediator for parasite egression from parasitophorous vacuoles. Production of phosphatidic acid by diacylglycerol kinase 2 on the vacuolar outside leads to activation of an atypical GC which in turn activates PKG (Bisio et al. 2019). Whether PKG from *Toxoplasma gondii* is a suitable drug target to prevent parasite reproduction and thus Toxoplasmosis has still to be evaluated.

## Outlook

The 9th international cGMP conference provided an in depth overview on all aspects of cGMP research in a very focused and fruitful research atmosphere. Highlights of this conference were definitely the disclosure of the sGC structure and also seeing the broad application potential for cGMP-based drugs in various diseases. In the future, additional evidence from genetic modifications of sGC and pGC signaling as in cGMP degradation pathways which is driven by advanced sequencing technologies and GWAS studies will further enrich the field. This will broaden our understanding of the disease relevance of these cGMP pathways which will hopefully profit the patient. Therefore, the authors look very much forward to the upcoming cGMP meeting in 2021 which will be the 10th anniversary meeting of the conference, and will further extend our insights in the unique cGMP signaling. This meeting will be held in the city of Augsburg in Germany from June 18th to 20th in 2021.

## Abbreviations

AC: Adenylyl cyclase
AD: Alzheimer’s disease
ANP: Atrial natriuretic peptide
BNP: Brain natriuretic peptide
BPH: Benign prostatic hyperplasia
cAMP: Cyclic adenosine monophosphate
cCMP: Cyclic cytidine monophosphate
cGK: cGMP-dependent protein kinase
cGMP: Cyclic guanosine monophosphate
CNG: Cyclic nucleotide-gated ion channels
cNMP: Cyclic nucleotides
CNP: C-type natriuretic peptide
CNS: Central nervous system
CO: Carbon monoxide
CTEPH: Chronic thromboembolic hypertension
cUMP: Cyclic uridine monophosphate
CYB5R3: Cytochrome B5 reductase 3
Cys: Cysteine
DMD: Duchenne muscular dystrophy
ED: Erectile dysfunction
FRET: Fluorescence resonance energy transfer
GC: Guanylyl cyclase
GC-A: Guanylyl cyclase A
GC-B: Guanylyl cyclase A
GMP: Guanosine monophosphate
GTP: Guanosine triphosphate
HF: Heart failure
HFpEF: Heart failure with preserved ejection fraction
HFrEF: Heart failure with reduced ejection fraction
HTN: Hypertension
IIP: Idiopathic interstitial pneumonia
KO: Knockout
LPS: Lipopolysaccharide
LV: Left ventricle
mRSS: Modified Rodnan skin score
NEP: Neutral endopeptidase
NO: Nitric oxide
NOS: Nitric oxide synthase
NP: Natriuretic peptide
NPR1: Natriuretic peptide receptor 1
NPR2: Natriuretic peptide receptor 2
NPR-A: Natriuretic peptide receptor A
NPR-B: Natriuretic peptide receptor B
PAH: Pulmonary arterial hypertension
PDE: Phosphodiesterase
PfPKG: *Plasmodium falciparum* PKG
pGC: Particulate guanylyl cyclase
PH: Pulmonary hypertension
PH-LVD: Left ventricular disease
PKG: Protein kinase G
RBC: Red blood cells
SCD: Sickle cell disease
sGC: Soluble guanylyl cyclase
SMC: Smooth muscle cell
SSc: Systemic sclerosis
T2D: Type two diabetes
VASP: Vasodilator-stimulated phosphoprotein
VSMC: Vascular smooth muscle cell
WT: Wild-type
